# Applying Methods for Postnatal Growth Assessment in the Clinical Setting: Evaluation in a Longitudinal Cohort of Very Preterm Infants

**DOI:** 10.3390/nu11112772

**Published:** 2019-11-14

**Authors:** Montserrat Izquierdo Renau, Victoria Aldecoa-Bilbao, Carla Balcells Esponera, Beatriz del Rey Hurtado de Mendoza, Martin Iriondo Sanz, Isabel Iglesias-Platas

**Affiliations:** 1 Neonatology Department, Hospital Sant Joan de Déu, Institut de Recerca Sant Joan de Déu, Universidad de Barcelona, BCNatal, Esplugues de Llobregat, 08950 Barcelona, Spain; cbalcells@sjdhospitalbarcelona.org (C.B.E.); bdelrey@sjdhospitalbarcelona.org (B.d.R.H.d.M.); miriondo@sjdhospitalbarcelona.org (M.I.S.); iiglesias@sjdhospitalbarcelona.org (I.I.-P.); 2 Neonatology Department, Hospital Clinic, Universidad de Barcelona, BCNatal, 08028 Barcelona, Spain; valdecoa@clinic.cat

**Keywords:** postnatal growth failure, extrauterine growth restriction, intrauterine growth restriction, fall in *z*-score

## Abstract

Aim: To analyze different methods to assess postnatal growth in a cohort of very premature infants (VPI) in a clinical setting and identify potential early markers of growth failure. Methods: Study of growth determinants in VPI (≤32 weeks) during hospital stay. Nutritional intakes and clinical evolution were recorded. Growth velocity (GV: g/kg/day), extrauterine growth restriction (%) (EUGR: weight < 10th centile, *z*-score < −1.28) and postnatal growth failure (PGF: fall in *z*-score > 1.34) at 36 weeks postmenstrual age (PMA) were calculated. Associations between growth and clinical or nutritional variables were explored (linear and logistic regression). Results: Sample: 197 VPI. GV in IUGR patients was higher than in non-IUGRs (28 days of life and discharge). At 36 weeks PMA 66.0% of VPIs, including all but one of the IUGR patients, were EUGR. Prevalence of PGF at the same time was 67.4% (IUGR patients: 48.1%; non-IUGRs: 70.5% (*p* = 0.022)). Variables related to PGF at 36 weeks PMA were initial weight loss (%), need for oxygen and lower parenteral lipids in the first week. Conclusions: The analysis of *z*-scores was better suited to identify postnatal growth faltering. PGF could be reduced by minimising initial weight loss and assuring adequate nutrition in patients at risk.

## 1. Introduction

Growth of preterm infants lacks a standardized approach among neonatologists [1,2] and a multitude of methods have been described to report growth trajectories in these patients. Calculating growth velocity (GV) as g/kg/day [3,4] is one of the most widely used, followed by differences in *z*-score between two points in time [5]. A recent review and meta-analysis [6] examined the variability of growth assessment regarding calculations, time frames, and denominators, as well as how extrauterine growth restriction (EUGR) was defined. It concluded that the most frequent method to calculate GV was g/kg/d, followed by g/d and change in *z*-score relative to an intrauterine or postnatal growth chart. Most studies calculated g/kg/d using birth/admission as the baseline, followed by a smaller percentage using other time points like time at full enteral feedings or the day after regaining birth weight (BW). Some authors have explored theoretical mathematical models to individually approach growth velocity taking into account the initial period of weight loss [7,8]. Although promising, they have not been fully implemented in clinical practice yet.

On the other hand, there is an ongoing debate focused on the ideal growth pattern for preterm infants or, in other words, which curves should be used to monitor preterm growth [9,10,11,12]. The most largely accepted recommendation is that growth of a preterm baby should match that of a healthy fetus, as the American Academy of Pediatrics reported in 1977 [13], but we still lack a globally accepted reference for weight, length, head circumference or body composition [1,14]. In our NICUs, this target is not always attained, with EUGR being a very common issue [15,16,17] although several authors have reported growth close to fetal references after enhanced nutritional policies [18,19,20]. The use of longitudinal postnatal growth curves rather than cross-sectional size-at-birth by gestational age (GA) reference curves has been advocated by some authors [14,21]. They support the idea that fetal growth depends on the intrauterine environment and placental function, while a preterm infant is under the influence of different factors such as nutritional support and clinical conditions [22]. There are no studies comparing long-term outcomes of well-grown and growth-restricted babies classified by different curves, so there is little evidence to support which reference would be optimal.

Both postnatal growth and the quality of early nutritional support of very preterm infants (VPI) have been recognized as capital factors contributing to progression in hospital and after discharge, with nutritional deficiencies and faltering growth during admission having been related to worse short- and long- term outcomes [23]. Some authors have reported that faltering growth specially affects the sickest patients, due to the mixed effect of inflammatory mediators on metabolism and the difficulty in achieving recommended nutritional intakes due to fluid restriction [24,25]. In a large cohort of preterm infants, Ehrenkranz showed that this relationship is not unidirectional and that the risk of adverse outcomes was influenced by early energy intake [26]. Improved growth and nutritional supply during admission have been related to better long-term growth of brain structures and a more favorable neurodevelopmental outcome [27,28,29,30,31]. On the other hand, very rapid postnatal catch-up growth after a period of growth restriction has been linked to adverse metabolic consequences [32,33,34]. In this regard, preventing postnatal growth faltering would avoid the need for catch up growth. In daily practice, we are responsible for providing our patients with the optimal nutrient supply in order to reach a positive weight gain and prevent postnatal growth faltering, but this confusing scenario of how growth should be evaluated and reported, leaves the clinical neonatologist very poorly equipped to face this challenge.

The aims of this study were: (1) to describe the applicability of the most currently used methods for the assessment of postnatal growth in clinical practice using an actual cohort of VPI, (2) to pinpoint early markers that can help detect which children, conditions and macronutrient provision profiles associate the highest risk of postnatal growth failure in order to provide some room for timely intervention.

## 2. Materials and Methods

### 2.1. Study Design

Prospective cohort study of determinants of growth in VPI admitted in our unit between 2011 and 2016. This analysis includes growth data from the participants of a study aimed to search for different markers of safety and efficacy of nutritional support in very preterm babies (PIC-95-13). The protocol was approved by the local ethics committee and written consent from the parents was obtained prior to participation.

### 2.2. Study Population

Newborns with a gestational age equal or under 32 weeks that were admitted within 24 h of birth were eligible for the study. Exclusion criteria were death before one week of life, major malformations, chromosomal abnormalities or genetic diseases and congenital infection.

### 2.3. Growth Data

Prenatal diagnosis of intrauterine growth restriction (IUGR) was established following local obstetrics protocols if estimated fetal weight was under the 3rd centile or under the 10th centile with abnormal doppler waves [35]. Postnatal weight was recorded daily from birth and length and head circumference were determined weekly during admission by the clinical staff in charge of care. Only longitudinal weight measurements were evaluated in this study. Raw data were transformed into *z*-scores in reference to local intrauterine curves for comparison with the population of the same gestational or postmenstrual age (PMA) at each point [36]. Post discharge weight data for children that were discharged before 36 weeks PMA was available from the home hospitalization program. Growth velocity (GV g/kg/day) was calculated as 2-point birth weight model (GV = (1000 × (Wn − W1))/W1 × (Dn − D1)) where W is the weight in grams, D is day, 1 indicates the beginning of the time interval, and n is the end of the time interval in days [3]. We did not consider the logarithmic method [3] suitable to be readily available in clinical practice. For consistency with the literature, we defined EUGR at any time point if *z*-score was under −1.28 (10th centile) [37]. A fall in *z*-score (FZS) by more than 1.34 between two points was termed Postnatal Growth Failure (PGF). We selected a cut-off point double the one described for catch-down growth for term infants since growth failure is much more prevalent in preterm infants [38]. Regarding initial weight loss, data were collected on minimum weight, days to maximum weight loss and days to regain BW.

### 2.4. Nutritional Protocols and Nutritional Variables

Parenteral nutrition (PN) was started at admission in all patients. Our parenteral nutrition protocol recommends an initial composition of 8.6 g/kg carbohydrates, 2.5 g/kg proteins and 2 g/kg lipids followed by a stepwise increase of nutrients during the first week of life to a maximum of 17 g/kg carbohydrates, 3.5–4 g/kg proteins and 3–3.5 g/kg lipids at 7 days of life. Enteral feeds followed as soon as possible depending on patient stability in the form of human milk (own’s mother first, alternatively donor) Following our protocol, donor milk was available for the first 28 days of life in preterm infants ≤28 weeks or for the first week of life for those >28 weeks and aimed to be increased daily by 20–30 mL/kg to a maximum of 160–180 mL/kg/day. Babies that required fluid restriction due to medical conditions were limited to 145–155 mL/kg/day. Fortification was started with Pre-nan FM-85^®^(Nestle^®^) when an intake of 100 mL/kg/day of human milk was attained. We defined the day to achieve full enteral feeds as the first day without intravenous nutrition. Actual macronutrient intakes and energy were calculated from the administered volumes of PN considering daily macronutrient PN prescription and milk registered in nursing charts. For commercial formula, composition was extracted from manufacturer’s information; calculations for breast milk were based on reference values [39]. Nutrients and energy supplied by human milk fortifier were calculated assuming a standard composition of 1 mL of fortified human milk taken from manufacturer’s information (1 g fortifier per 25 mL of human milk).

### 2.5. Clinical Outcomes

A diagnosis of Patent Ductus Arteriosus (PDA) was assigned in the presence of a compatible heart murmur or clinical signs (hyperdynamic precordial impulse, full pulses, widened pulse pressure, and/or worsening of the respiratory status) with a ductal right-to-left shunt in the echocardiography. Babies needing oxygen for more than 28 days were categorized as having bronchopulmonary displasia (BPD) [40]. Necrotizing enterocolitis was defined using Bell’s classification and diagnosed if a stage 2 or more was present [41]. Retinopathy of prematurity (ROP) was classified according to the International Committee for classification of ROP [42]. Intraventricular hemorrhage (IVH) was graded according to the Papille classification [43].

### 2.6. Statistical Analysis

Descriptive statistics are reported in absolute (n) and relative (%) frequencies and mean ± standard deviation (SD) or medians with 25th and 75th percentiles. Appropriate statistical tests (Student´s *t*-test for continuous variables and χ^2^ for categorical variables or Fisher exact test and Mann–Whitney *U* test as applicable) were used to evaluate the differences between groups. The analysis of the association between growth (expressed as mean weight *z*-score change or as postnatal growth failure) and all relevant clinical variables were conducted using linear or logistic regression models. The impact of PN on growth in the first 28 days of life was adjusted for gestational age and expressed as the β coefficient with 95% Confidence Interval. Predictors of PGF are presented as odds ratios (OR) and 95% confidence interval. Variables related to PGF with *p* < 0.10 in bivariate analysis were considered for inclusion in the multivariate analysis. Finally, we used a backward stepwise regression to select the predictors of PGF in the fitting model. Measures to assess variation (R^2^), calibration (Hosmer-Lemeshow “goodness-of-fit” test) and discrimination (area under the receiver operating characteristic curve (AUC)) were calculated to evaluate the performance of the predictive model. Statistical significance was set at two-sided *p* < 0.05. All analyses were performed by using IBM SPSS statistical software, version 22 (IBM Corp, Armonk, NY, USA).

## 3. Results

### 3.1. Description of the Sample

Over the study period, 209 preterm neonates ≤32 weeks were recruited Twelve had to be excluded due to early death (*n* = 1), diagnosis of associated conditions (*n* = 9; 1 CMV congenital infection, 5 major malformations- 3 cardiac malformations, 1 oesophageal and 1 jejunal atresia- and 3 chromosomal/ genetic abnormalities) or parental decision to withdraw (*n* = 2). The remaining 197 had a mean GA at birth of 29.0 ± 2.3 weeks and BW 1200 ± 360 g. Half of the sample (*n* = 104, 52.8%) were males. Most mothers received at least one dose of antenatal steroids (*n* = 173, 87.8%) and 105 of the babies (53.3%) presented respiratory failure at some point during admission needing oxygen therapy. Twenty-seven patients (13.7%) had been diagnosed of IUGR. Almost three quarters of the sample, (*n* = 143, 74.9%) were discharged home after 36 weeks PMA. Six patients (3%) died during the study period (after 1 week of life).

### 3.2. Growth Velocity (g/kg/day)

The mean 2-point weight GV until day 28 of life was 10.4 ± 4.7 g/kg/day and 16.1 ± 5.8 g/kg/day from birth to discharge. Maximum percentage of initial weight loss was 8.3 ± 4.6%. 2-point weight GV until day 28 of life was positively correlated with increasing gestational age, but the correlation was negative for GV until discharge (Table 1). 2-point weight GV in IUGR patients was higher than in non-IUGR patients both at 28 days of life (12.9 ± 4.6 vs. 9.9 ± 4.6, *p* = 0.005) and from birth to discharge (18.7 ± 7.2 vs. 15.4 ± 6.0, *p* = 0.03).

### 3.3. EUGR (z-Score < −1.28)

The prevalence of EUGR in the whole sample was 66.0% (*n* = 126) at 36 weeks PMA and 56.5% at discharge (*n* = 108). All IUGR patients but one were EUGR at both 36 weeks PMA and discharge, while among non-IUGRs, we found a 61.0% (*n* = 100) prevalence of EUGR at 36 weeks and 49.4% (*n* = 81) at discharge.

### 3.4. Fall in z-Score (FZS > 1.34)

We found a significant inverse lineal trend between GA and weight FZS (mean weight FZS −1.0 ± 0.5 at 28 days and −1.7 ± 0.8 at 36 weeks) (Table 1). Two thirds of weight FZS at 36 weeks occurred in the first 28 day of life, with a 0.34 ± 0.13 mean decrease in weight *z*-score for every 10 days of life before reaching 36 weeks of PMA. Mean FZS at discharge was −1.5 ± 0.7 for weight, −1.2 ± 1.1 for length and −0.7 ± 1.1 for head circumference. Two thirds (*n* = 130, 67.4%) of patients had PGF at 36 weeks PMA (67.4%). The prevalence was 48.1% among IUGR patients and 70.5% in non-IUGRs (*p* = 0.022).

### 3.5. Early Predictors of Postnatal Growth Failure at 36 Weeks Postmenstrual Age

We explored different potential predictors of PGF at 36 weeks PMA. The single major determinant of PGF in univariate analysis was GA at birth, OR = 0.77 (CI95% 0.66–0.90); *p* = 0.001. 

Regarding demographic and perinatal characteristics upon admission, patients that developed PGF at 36 weeks PMA were more immature, had a lower incidence of IUGR and in most cases needed surfactant therapy at birth (Table 2).

During admission, preterm infants that developed PGF at 36 weeks PMA required more days on supplementary oxygen, mechanical and non-invasive ventilation and antibiotics and had a higher incidence of complications (PDA, IVH, ROP and late onset infections) and a longer length of stay in intensive care (Table 3).

When we analysed the nutritional practices in both groups, we did not find significant differences between the nutritional intake (parenteral and enteral macronutrients and fluids) in the first 28 days of life (see Supplementary Table S1). PGF babies had a lower weight GV (g/kg/day) between birth and 28 days of life (8.6 ± 4.0 vs. 13.8 ± 5.0; *p* < 0.001). Their percentage of initial weight loss was higher (9.4 ± 5.0% vs. 6.3 ± 4.0%; *p* < 0.001) and they took longer to regain BW (11.3 ± 5 vs. 8.8 ± 5 days; *p* < 0.001). We saw no differences in time to achieve full enteral feeds (12.9 ± 7.0 vs. 12.9 ± 13.6 days, *p* = 0.986).

In the analysis of nutritional determinants of growth, we identified protein and lipid content as well as protein to energy ratio in PN during the first week of life as limiting factors of the FZS at 14 and 28 days of life (Table 4). Also, of note, the most immature patients received a greater intake of lipids and proteins in PN and a lower volume of enteral nutrition during the first week, received PN for longer and needed more time to achieve full enteral feeds (Table 1). 

We performed a multivariate analysis of clinical and nutritional early predictors of PGF at 36 weeks PMA by logistic regression (Table 5). Finally, after backward stepwise regression, four variables were kept in the model (*R*^2^ = 0.39, *p* < 0.001): GA (OR = 0.72 (CI95% 0.58–0.90); *p* = 0.003), maximum percentage of BW loss (OR = 1.31 (CI95% 1.17–1.46); *p* < 0.001), oxygen during admission (OR = 5.00 (CI95% 2.10–11.90); *p* < 0.001) and lipids in PN in the first week of life (OR = 0.47 (CI95% 0.25–0.88); *p* = 0.019. We found a good accuracy of the predictive model with an AUC = 0.82 (CI95% 0.76–0.88); *p* < 0.001.

## 4. Discussion

Our principal aim was to explore different methods to describe growth trajectories and to define growth failure during admission from a practical and clinical point of view. For this, we analysed an actual prospective cohort of VPI. In a second step, we have looked at factors related to PGF at 36 weeks PMA, finding that besides GA, a greater maximum percentage of initial weight loss, the need of oxygen supply during admission and a lower provision of lipids during the first week of life were additional independent risk factors.

In our cohort, GV (g/kg/day) calculated by the 2-point method between birth and discharge is inversely proportional to GA, giving the factitial effect that growth is better in the most immature infants. This is due to the presence of a smaller denominator (BW) and the effect is similar for those babies who are growth restricted at birth, as reported by other authors [17,44]. Longer lengths of stay also influence the final calculation of GV by this method, further distorting interpretation for the most vulnerable infants, who spent more time in hospital [3]. This is illustrated by the fact that if a single homogeneous time point (i.e., 28 days of life) is chosen instead, GV by the 2-point method correctly reflects that more mature babies grow better (see Table 1). Age at discharge has a wide variability, ranging in our sample from a minimum of 17 days to a maximum of 162 days. The exponential method to calculate GV has been advocated as one of the most suitable, especially in research settings [1] since it is not affected by BW or length of stay, but its mathematical calculation for daily clinical application is not as straightforward as the 2-point method [3,4]. One alternative could be the use of the 2-point average weight method for patient evaluation in the unit [4,19], although its value is still limited because, as all GV methods, it does not account for gender or postmenstrual age. We chose the 2-point method for our analysis because is still one of the most used in daily practice and we aimed to emphasize how misleading it can be when used for growth calculations and nutritional prescriptions.

The WHO recommends *z*-scores as the best way to present anthropometric data [45] and since 2005 reporting change in *z*-scores seems more widely adopted [6]. EUGR was defined as a *z*-score of weight below the 10th centile, as in other series, and consistent with their results [17,46], growth faltering during admission was remarkably prevalent in our sample with 66.0% EUGR at 36 weeks PMA and 56.5% at discharge. Defining a centile cut-off point to define growth restriction implies that the presence of IUGR and the *z*-score of BW will have a huge impact in the final categorization. In fact, only 1 (3.7%) of the IUGR patients was non-EUGR at 36 weeks in our series. Prevalence of EUGR is rarely reported separately for preterms that were IUGR or normally grown at birth [17,47,48].

In our population, using PGF (change or FZS) instead of weight below 10th centile, better reflects the specific effect of postnatal growth independently of BW or BW *z*-score, while adjusting for GA and sex. Even so, there is also a difference in the proportion of IUGR and non-IUGRs affected by PGF at 36 weeks PMA (48.1% and 70.5%, respectively), probably reflecting that the biggest component of growth failure in IUGR is prenatal. Our choice of cut-off point of −1.34 FZS to define PGF can be debatable. It represents double the change described for catch-up growth [38]. Other authors have used a change in *z*-score > 1.28, probably using the number that represents the 10th centile in a distribution [5,47]. Rochow et al. [7], stated that in a healthy cohort of preterm infants born before 34 weeks GA, a FZS near 1 was due to the physiological early weight loss, so a FZS below that point could be considered growth faltering. The standard deviation for FZS was about 0.2, so that a loss of median + 2SD would also fall in the range of our selected cut-off. When analyzing the “healthy” subgroup of our patients, the median of FZS at 36 weeks PMA is −1.37, quite close to −1.34.

When assessing growth by differences in *z*-score, reference to the growth curves used is essential for the clinician and for the consistency of comparisons. For our study we selected local size-at-birth reference curves [36] in current use in our unit when the participants were admitted, in line with our aim to reflect the practicalities of real of growth evaluation in the clinical unit. Nevertheless, their agreement with the Fenton curves using Passing-Bablok regression (data not shown) was good, and although there were differences on calculated *z*-scores at certain time points, the FZS did not change when using one or the other as a reference. The adequacy of using cross-sectional growth references or the standard prescriptive preterm postnatal longitudinal curves launched by the Intergrowt-21st (ITG-21st) consortium [14,21] is still a matter of debate. Some authors have published comparative analysis between ITG-21st preterm size at birth reference and postnatal growth standards for preterm infants with local customized growth curves or with other widely accepted references like the Fenton curves [49,50,51,52], but there is still a lack of consensus due to lack of studies on the impact of growth categorization with different references on other clinical outcomes.

As in other reports [46,53], the greater percentage of weight loss or FZS in our sample happened before 28 days of life (two thirds of the total FZS). Physiological postnatal weight loss after extracellular space contraction and unmet higher nutritional needs due to severity of illness could be related to this clinical observation.

When exploring risk factors for PGF, GA was the single most relevant determinant. The most immature patients (born 23^0^–24^6^) showed the worst growth outcomes (PGF reaching almost 100% at 36 weeks PMA in this group), and we found an inverse linear trend for a decreasing prevalence of PGF with increasing GA (see Table 1). This finding is in agreement with previous studies [46,54]. This tendency is frequently thought to respond to higher severity of illness, but in a selected cohort of preterm infants considered healthy (both maternal and neonatal course free of complications) those born at earlier GAs also experienced a greater FZS in comparison to those of greater GAs [7], highlighting that either nutritional deficits or other factors must also play a role.

Patients that went on to develop PGF at 36 weeks PMA had a higher BW *z*-score and were less IUGR (Table 2). This is a common finding in the rare cases where growth results of SGA or IUGR preterms are specifically analysed. Severe and mild PGF (defined as FZS) seem less prevalent in those babies born SGA, and severe PGF is independently associated with a higher BW *z*-score, as shown by Shlomain (adjusted OR 3.17 for every unit increase) [54]. IUGR infants are born after a period of metabolic adaptation leading to impaired growth in utero, and they experience different growth patterns after birth, showing less physiological weight loss [55]. How to feed these patients and monitor their growth to avoid complications at short and long term remains unclear [56,57].

Those patients exhibiting PGF in our cohort had worse clinical outcomes during admission (see Table 3) and spent more days in the NICU. Other authors have reported the same results in large population studies [47,58,59]. Higher initial weight loss might be an early indicator of the risk of PGF at 36 weeks PMA, as others have reported [60]. In contrast to other studies [44,47,61], we saw no differences between groups in the time to reach full enteral feeding.

Enhanced early nutrition has been proven to decrease growth failure during admission [62,63,64,65,66]. In our case, we found no differences in macronutrient intake during the first month of life in infants with PGF, but greater parenteral protein and lipid supply during the first week seemed to reduce FZS at 14 and 28 days of life. Energy and nutrition utilization in VPI is a very complex process influenced also by intercurrent morbidities. Actual energy and protein intakes in our population might have been enough to sustain growth in stable infants but not in the critically ill ones. Patients with PGF are usually the sickest, which makes their energy expenditure higher and they undergo many stressful and inflammatory conditions that impact the capacity for nutrient absorption and consumption [25,67,68].

After multivariate analysis, the significant predictors of PGF at 36 weeks were GA, which is unmodifiable, and a greater maximum percentage of initial weight loss, the need for oxygen therapy during admission and a lower provision of lipids during the first week of life. This is in agreement with the conclusions in another cohort that included neonates that were slightly more mature and with a higher prevalence of IUGR [69], with the exception that they did not include initial weight loss in their analysis [69].

One of the strengths of our study is the homogeneity of care, due to the single-center prospective design with unchanged clinical and nutritional protocols during the study period. Also, few studies have focused on how theoretical approaches of analysing growth in a real preterm cohort can be integrated into daily NICU work and impact their clinical relevance. One of the weaknesses of our study is that growth assessment was only based on weight, without considering length or head circumference. Although we did record these measurements, they were far less accurate than weight. Also, most discussions about preterm growth in the literature focus on weight, so this parameter was selected to model how different approaches perform in an actual very preterm cohort. Nevertheless, we do recognise that length, head circumference and probably additional tools should be evaluated in order to assess growth as a whole in preterm patients. Our participating cohort is now being followed up with a focus on neurodevelopment and metabolic health, so there should be an opportunity to further assess which methods for evaluating growth during admission can better predict future outcomes.

Our study has explored several methods to describe growth in an actual cohort of VPI, showing that FZS is currently the most meaningful for daily clinical practice, even though which reference curves and cut-off points should be used is still a matter of investigation by different groups. We have described some early predictors of postnatal growth faltering that can help clinicians when caring for the most vulnerable patients. Further research is needed to allow scientific societies to make a stronger recommendation of which method of evaluation of preterm growth during admission will lead to improvement in metabolic health and neurodevelopment in these patients.

## Figures and Tables

**Table 1:** Growth patterns and nutritional support according to GA at birth.

	Gestational Age (weeks)					
	23–24.6 (*n* = 14)	25–26.6 (*n* = 31)	27–28.6 (*n* = 40)	29–30.6 (*n* = 68)	31–32.0 (*n* = 44)	*p*-Value
**Weight *z*-score**						
Birth	−0.39 ± 0.75	0.21 ± 1.02	−0.09 ± 1.33	−0.02 ± 0.71	−0.21 ± 0.85	0.248
14 days	−1.22 ± 0.58	−0.74 ± 0.68	−0.72 ± 0.88	−0.83 ± 0.55	−1.19 ± 0.72	0.005
28 days	−1.41 ± 0.62	−0.92 ± 0.57	−0.99 ± 0.84	−1.12 ± 0.63	−1.51 ± 0.66	0.002
36 weeks	−2.42 ± 0.63	−1.75 ± 0.74	−1.84 ± 1.03	−1.46 ± 0.64	−1.49 ± 0.75	<0.001 *
**Nutrition first week**						
Parenteral Protein (g/kg/day)	2.8 (2.6–3.1)	2.9 (2.7–3.2)	2.9 (2.6–3.0)	2.6 (2.2–2.9)	2.2 (1.6–2.8)	<0.001 *
Parenteral Carbohydrates (g/kg/day)	8.3 (7.1–9.6)	9.9 (8.8–10.5)	9.6 (9.0–10.6)	9.8 (8.6–10.7)	9.2 (7.4–10.5)	0.006
Parenteral Lipids (g/kg/day)	2.3 (1.9–2.5)	2.3 (2.1–2.6)	2.1 (1.8–2.4)	1.8 (1.3–2.2)	1.4 (0.9–2.0)	<0.001 *
Protein/100 kcal ratio	4.0 (3.7–4.3)	3.9 (3.7–4.1)	3.8 (3.5–3.9)	3.6 (3.3–3.8)	3.4 (3.1–3.7)	<0.001 *
Average milk intake (mL/kg/day)	9.6 ± 6.1	14.7 ± 11.1	17.8 ± 15.3	29.8 ± 17.6	31.8 ± 19.0	<0.001 *
**Weight gain and enteral tolerance (0–28 days)**						
2-point weight GV * (g/kg/day)	8.0 ± 3.7	9.3 ± 5.0	9.8 ± 5.9	11.1 ± 3.8	11.3 ± 4.6	0.083 *
Maximum % weight loss	5.8 ± 3.4	8.2 ± 5.3	8.5 ± 5.5	8.9 ± 4.4	8.2 ± 3.2	0.312
DOL to regain birth weight	9.5 (5.8–12.5)	8.5 (6.8–12)	9 (7–16)	10 (8–12)	10 (7–12)	0.256
DOL full enteral feeds	13.5 (12–22.5)	13.0 (11–18.5)	12 (10–20.5)	9 (7.3–11.8)	8 (7–10)	<0.001 *
PN (days)	27.5 (15–37)	13 (11–23)	12.5 (9–21)	8 (7–11)	8 (6–9)	<0.001 *
**Growth from birth to discharge**						
**Fall in *z*-scores**						
Weight	−1.54 ± 0.92	−1.82 ± 0.89	−1.72 ± 0.77	−1.34 ± 0.51	−1.06 ± 0.48	<0.001 *
Length	−2.16 ± 1.17	−1.72 ± 1.44	−1.62 ± 1.12	−1.08 ± 0.81	−0.69 ± 0.93	<0.001 *
Head circumference	−0.26 ± 1.0	−0.57 ± 1.36	−0.38 ± 1.05	−0.88 ± 1.19	−0.51 ± 0.80	0.125
**Growth velocity (g/kg/day)**						
2-point weight GV (birth-discharge)	26.5 ± 4.9	20.2 ± 4.5	17.8 ± 5.7	13.8 ± 3.4	12.6 ± 4.2	<0.001 *
EUGR at 36 weeks (%)	100	72.0	69.7	48.3	48.6	<0.001 *
EUGR at discharge (%)	78.6	57.1	63.6	41.7	28.6	<0.001 *
**Fall in weight *z*-score**						
Birth-14 days	−0.84 ± 0.50	−0.95 ± 0.64	−0.63 ± 0.66	−0.80 ± 0.40	−0.98 ± 0.33	0.022
Birth-28 days	−1.02 ± 0.46	−1.08 ± 0.71	−0.89 ± 0.68	−1.07 ± 0.38	−1.09 ± 0.43	0.459
Birth-36 weeks	−2.05 ± 0.73	−1.92 ± 0.98	−1.75 ± 0.68	−1.44 ± 0.38	−1.27 ± 0.43	<0.001 *
PGF-36 weeks (%)	92.3	82.1	72.5	63.2	52.3	0.017 *

* Linear Trend *p*-value < 0.01. Values are number (%), median (p25–p75) or mean ± standard deviation. Abbreviations. DOL = Day of life. EUGR = Extrauterine Growth Restriction. PGF = Postnatal growth failure. PN = Parenteral Nutrition.

**Table 2:** Initial demographics and perinatal characteristics according to growth status at 36 weeks PMA.

	Postnatal Growth Failure		*p*-Value
	Yes (*n* = 130)	No (*n* = 63)	
Birth gestational age (weeks)	28.7 ± 2.4	29.9 ± 1.8	<0.001
Birth weight (grams)	1218 ± 368	1186 ± 344	0.559
Birth weight *z*-score (SD) Ɨ	0.21 ± 0.8	−0.66 ± 0.9	<0.001
Male gender	68 (52.3)	33 (52.4)	0.992
IUGR	13 (10.0)	14 (22.2)	0.022
Antenatal steroids (≥1)	112 (86.2)	57 (90.5)	0.394
Caesarean section	86 (66.2)	45 (71.4)	0.462
Multiple birth	43 (33.1)	24 (38.1)	0.492
Apgar at 5 min (<6)	10 (7.8)	4 (6.3)	0.726
Surfactant administration	63 (48.5)	14 (22.2)	<0.001
Early sepsis	2 (1.5)	0 (0)	0.322
Temperature at admission (°C)	36.4 ± 0.7	36.3 ± 0.7	0.771

Values are number (%) or mean ± standard deviation. Ɨ Birth weight *z*-score according to local charts [36]. Abbreviations. IUGR = Intrauterine Growth Restriction.

**Table 3:** Main outcomes according to postnatal growth status at 36 weeks PMA.

	Postnatal Growth Failure		*p*-Value
	Yes (*n* = 130)	No (*n* = 63)	
MV (days)	6.1 ± 13	2.5 ± 7	0.018
Oxygen (days)	22.8 ± 37	10.4 ± 27	0.009
NIV (days)	27.6 ± 26	18.5 ± 23	0.018
PN (days)	14.1 ± 12	13.2 ± 16	0.663
Insulin therapy first week	18 (13.8)	5 (7.9)	0.235
Antibiotics (days)	13.3 ± 16	8.2 ± 11	0.009
Diuretics (days)	18.4 ± 29	7.3 ± 19	0.002
Central line catheter (days)	14.3 ± 11	13.4 ± 15	0.654
NICU days	36.3 ± 34	24.4 ± 28	0.011
PDA	61 (46.9)	19 (30.2)	0.027
Surgical PDA	11 (8.5)	3 (4.8)	0.353
ROP	46 (37.4)	12 (21.1)	0.029
ROP > 2 or plus disease	5 (4.1)	2 (3.5)	0.857
IVH	32 (24.6)	5 (7.9)	0.006
IVH > 2	7 (5.4)	2 (3.2)	0.495
NEC > 2	4 (3.1)	3 (4.8)	0.557
BPD	38 (29.5)	8 (12.9)	0.012
LOS	37 (28.5)	6 (9.5)	0.003
Length of stay (days)	66 ± 32	55 ± 28	0.021

Values are number (%) or mean ± standard deviation. Abbreviations: BPD = Bronchopulmonary dysplasia, IVH = Intraventricular haemorrhage, LOS = Late onset sepsis, MV = Mechanical ventilation, NEC = Necrotizing enterocolitis, NICU = Neonatal intensive care unit, NIV = Non-invasive ventilation, PDA = Patent ductus arteriosus, PN = Parenteral nutrition, ROP = Retinopathy of prematurity.

**Table 4:** Impact of PN * in the first week of life in FZS at 14 and 28 days adjusted for gestational age.

	Beta Coefficient for Fall in Weight *z*-Score Adjusted for Gestational Age (CI 95%)			
	At 14 days	*p*-Value	At 28 days	*p*-Value
Lipids	+0.20 (0.10–0.32)	0.002	+0.16 (0.02–0.31)	0.027
Proteins	+0.15 (0.19–0.27)	0.025	+0.12 (−0.04–0.28)	0.150
Carbohydrates	+0.01 (−0.04–0.05)	0.744	−0.01 (−0.06–0.04)	0.815
Protein/100 kcal ratio	+0.38 (0.02–0.56)	<0.001	+0.27 (0.05–0.49)	0.017

* Mean PN composition in the first week in the cohort (*n* = 193): protein 2.6 ± 0.6 g/kg/day; lipids 1.9 ± 0.6 g/kg/day, carbohydrates 9.4 ± 1.7 g/kg/day and energy 65 ± 13 kcal/kg/day. Abbreviations: PN = Parenteral Nutrition.

**Table 5:** Clinical and nutritional predictors evaluated at 28 days of postnatal growth failure at 36 weeks PMA (adjusted for birth gestational age).

	Predictors of PGF		
	Adjusted OR	95% CI	*p*-Value
IUGR	0.44	0.19–1.03	0.058
Oxygen during admission	2.61	1.29–5.26	0.008
Surfactant administration	2.57	1.26–5.25	0.010
Antibiotics (days)	1.01	0.97–1.04	0.695
PDA	0.70	0.35–1.39	0.305
IVH	0.37	0.13–1.05	0.062
LOS	0.45	0.16–1.27	0.131
Maximum % weight loss	1.25	1.14–1.38	<0.001
Time to regain birth weight	1.16	1.06–1.27	0.001
PN Lipids in the first week	0.59	0.34–1.01	0.055
PN Proteins in the first week	0.76	0.44–1.31	0.324
PN Protein/100 kcal in the first week	0.76	0.34–1.70	0.508

Adjusted for birth gestational age. Abbreviations: IUGR = Intrauterine Growth Restriction, IVH = Intraventricular haemorrhage, LOS = Late onset sepsis, PDA = Patent Ductus Arteriosus, PN = Parenteral Nutrition.

## Supplementary Materials

The following are available online at https://www.mdpi.com/2072-6643/11/11/2772/s1, Table S1: Postnatal growth failure according to nutritional intake (parenteral + enteral supply).
