# Supplementary material for: Applying Methods for Postnatal Growth Assessment in the Clinical Setting: Evaluation in a Longitudinal Cohort of Very Preterm Infants
Source: Nutrients. 2019 Nov 14;11(11):2772. doi: 10.3390/nu11112772 (PMC6893690; doi:10.3390/nu11112772)
Supplement: Supplementary file 1 [file nutrients-11-02772-s001.pdf]

Supplementary Table S1. Postnatal growth failure according to nutritional intake (parenteral+enteral supply).

	Postnatal Growth Failure		*p-value*
	Yes (n=130)	No (n=63)	
Volume (ml/kg/day)			
First week	103±12	105±11	0.190
Second week	141±12	144±15	0.203
At 28 days	155±24	161±20	0.145
Protein (g/kg/day)			
First week	3.0±0.5	3.0±0.4	0.829
Second week	3.3±0.6	3.4±0.7	0.781
At 28 days	4.0±1.3	4.0±1.0	0.152
Energy (kcal/kg/day)			
First week	82±9	83±9	0.304
Second week	109±18	114±17	0.057
At 28 days	130±24	133±21	0.364
Protein/100 Kcal ratio			
First week	3.7±0.5	3.6±0.4	0.252
Second week	3.1±0.4	3.0±0.5	0.118
